# Is There a Correlation Between Periodontal Disease Symptoms and the COVID-19 Vaccination?

**DOI:** 10.7759/cureus.58892

**Published:** 2024-04-24

**Authors:** Bann AlHazmi, Zuhair S. Natto, Mayson AlQarni

**Affiliations:** 1 Periodontics and Community Dentistry, King Saud University, Riyadh, SAU; 2 Dental Public Health and Periodontology, King Abdulaziz University, Jeddah, SAU

**Keywords:** oral malodor, tooth mobility, inflammation, covid-19 vaccines, gingival bleeding, periodontal disease

## Abstract

Objective: This study aimed to investigate and compare potential associations between different COVID-19 vaccines and periodontal diseases, mainly gingival bleeding and oral malodor (bad breath).

Materials and methods: This cross-sectional study used an online questionnaire consisting of 15 questions regarding demographic information, medical history, type of COVID-19 vaccine received, history of COVID-19, and general and periodontal symptoms after vaccination. The survey was voluntary and privately accessed online using SurveyMonkey^®^. A total of 2000 participants from three regions of Saudi Arabia participated in the study from October 11, 2021, to October 11, 2022.

Results: Of the participants, 95.8% received at least one dose of the available COVID-19 vaccine. Oxford-AstraZeneca was the most administered (41.7%). Patients who suffered from chronic diseases or had a history of COVID-19 infection were less likely to be vaccinated (OR= 0.62, 95% CI 0.40-0.97; OR=0.62, 95% CI 0.39-0.99) compared with people with no chronic diseases or who had a history of COVID-19. The odds ratios for gingival bleeding, oral malodor, mobility, and tooth loss indicated no significant differences regarding vaccination status.

Conclusion: COVID-19 vaccines might not affect periodontal tissue conditions. People should not avoid vaccination due to concerns with oral or general health, as the benefits of vaccination outweigh the potential side effects.

## Introduction

The ongoing battle against the novel severe acute respiratory syndrome coronavirus 2 (SARS-CoV-2) has been a rollercoaster of hope and despair. However, thanks to the development of vaccines, the fight against the pandemic has become easier, marking a significant breakthrough. The general population has seen a remarkable improvement in outcomes after COVID-19 infection, and various studies support the safety and efficacy of COVID-19 vaccines in such cases [1-3].

Several studies have revealed that COVID-19 is associated with the severity of periodontitis [4-8]. Moreover, COVID-19 can trigger cytokine storms that increase several inflammatory markers [2,3]. These markers have been associated with COVID-19 severity. Inflammatory markers, notably C-reactive protein (CRP), interleukin-6 (IL-6), and erythrocyte sedimentation rate (ESR), have a positive correlation with COVID-19 severity [2,3]. Recent studies suggest that COVID-19 may cause diseases such as pediatric inflammatory multisystemic syndrome, idiopathic thrombocytopenic purpura, rheumatic diseases, and autoimmune hemolytic anemia, possibly through molecular mimicry [9,10].

The effect of COVID-19 on oral health is often overlooked in the literature; however, it is crucial to consider its impact. Gingival bleeding, which is an early sign of gingivitis, has been linked to infection and inflammation [11,12]. Moreover, periodontal inflammation has been identified as a risk factor for systemic inflammation, including diabetes mellitus and cardiovascular disease [13,14]. Periodontal disease that is caused by specific bacteria triggers the production of inflammatory mediators, leading to the loss of tissue structure and function around the teeth [15].

Bad breath (oral malodor) is mainly an oral condition but is also associated with other infections and diseases. The primary causes of oral malodor are gingivitis, periodontitis, and tongue coating. Periodontal disease can increase the severity of malodor, but its association with oral malodor is still controversial [16].

While vaccines are effective in the general population, there are significant gaps in vaccine safety and efficacy data for vulnerable populations, such as those with systemic autoimmune and inflammatory disorders, those using immunosuppressive medications, and pregnant women. Ongoing studies continue to address these gaps to ensure that everyone has access to safe and effective vaccines [17,18]. In a recent study by Rosa et al., it was found that patients who had COVID-19 before vaccination presented with the worst periodontal condition (bleeding on probing, probing depth, and mean clinical attachment level) when compared to patients with no history of COVID-19. However, their sample size was based on 95 patients only and in a dental setting. They suggested having a larger sample size to confirm these findings [19].

Given these findings, the question of the impact of the COVID-19 vaccine on periodontal status has not been fully investigated. This study aimed to explore any potential associations between COVID-19 vaccinations and signs of periodontal diseases, mainly gingival bleeding and bad breath. Additionally, it sought to compare different types of COVID-19 vaccines as well regarding the general symptoms of periodontal disease. By shedding light on the impact of COVID-19 on oral health, this study can help establish public health policies and improve outcomes for vulnerable populations.

## Materials and methods

The present study was approved by the Institutional Review Board of the College of Medicine at King Saud University, Riyadh, Saudi Arabia (reference number, E-21-6199). Written informed consent was obtained from each participant before the survey to assure the privacy and secrecy of all registered data. This study complies with the ethical rules of the 64th World Medical Association Declaration, General Assembly, Helsinki (2013).

Participants

All Saudi residents above 18 years old who received the COVID-19 vaccine in Saudi Arabia were qualified to participate and included in the study. Patients who refused to participate were excluded.

Study design

This cross-sectional study included adults (above 18 years old) who received the COVID-19 vaccine (Oxford-AstraZeneca, Pfizer-BioNTech, Moderna, or mixed vaccines [combination of vaccines]) in Saudi Arabia in three regions within the largest population (Riyadh-Makkah-Eastern province). An online questionnaire (Appendix) was distributed through smartphones. Data were collected immediately after lockdown removal in Saudi Arabia; however, restrictions prevented direct contact between researchers and participants. The questionnaire consisted of 15 questions based on two previously validated articles conducted in Saudi Arabia on vaccines and COVID-19 [20,21]. The first one included demographic information, medical history, COVID-19 vaccine received, and history of COVID-19 [20], and the second one was about general and periodontal symptoms after vaccination [21]. The study was performed from October 11, 2021, to October 11, 2022.

Setting and application

The survey was completed within about 5 minutes for most participants. The voluntary and private survey, completed through SurveyMonkey®, was distributed to participants via a social media invitation. The participants were selected to represent the Saudi Arabian population during the COVID-19 pandemic in 2021. Participation was anonymous and performed entirely online, and the researchers could not personally identify the participants.

Sample size calculation

The sample size was determined using the calculator at the Survey System [28]. Based on a population of 30 million, with a confidence level of 95%, a power level of 80%, and a moderate effect size, 2000 was determined to sufficiently represent the vaccinated population in the three Saudi regions, as the Ministry of Health in Saudi Arabia initially aimed to vaccinate 70% of the population (about 28 million).

Statistical analysis

After data collection, the variables were encoded in an Excel spreadsheet using IBM Corp. Released 2015. IBM SPSS Statistics for Windows, Version 23.0. Armonk, NY: IBM Corp. Descriptive statistics were noted for certain variables, and chi-square was used to determine the relationship between several confounders (sex, age, marital status, educational level) and vaccination status at a significance level of 0.05. Logistic regression was used to assess the association between history of coronavirus infection, general symptoms, periodontal tissue conditions, and vaccination status regarding odds ratio (OR) and 95% confidence interval (CI).

## Results

A total of 2000 participants from different regions of Saudi Arabia were randomly selected to complete a survey on their mobile phones. We did not have any participants who refused to participate. 

General characteristics of the study sample

Table 1 shows the distribution of the study participants based on vaccination status. No potential confounders were different between the vaccinated and nonvaccinated groups. Patients who suffered from chronic diseases or had a history of COVID-19 infection were less likely to be vaccinated (OR= 0.62, 95% CI 0.40-0.97; OR=0.62, 95% CI 0.39-0.99) compared with people who have no history of COVID-19. Oxford-AstraZeneca was the most common COVID-19 vaccine (41.7%), followed by Pfizer-BioNTech (32.7%), a combination of vaccines (12.7%), and Moderna (8.8%) (Table 2).

**Table 1:** Distribution of the study participants according to their sex and age. * P-value < 0.05.

Variables	Total n (%)	Vaccinated n (%)	Non vaccinated n (%)	P-value
Sex				0.936
Male	866 (43.3)	827 (43.4)	36 (42.9)	-
Female	1134 (56.7)	1083 (56.7)	48 (57.1)	-
Age Group				0.062
Less than 30 years	359 (18)	349 (18.2)	10 (11.9)	-
30 to 40 years	641 (32.1)	618 (32.3)	23 (27.4)	-
41 to 50 years	609 (30.5)	578 (30.2)	28 (33.3)	-
51 to 60 years	283 (14.2)	263 (13.7)	20 (23.8)	-
Above 60 years	108 (5.4)	105 (5.5)	3 (3.6)	-
Marital status				0.269
Single	448 (22.4)	428 (22.4)	20 (23.8)	-
Married	1170 (58.5)	1126 (58.9)	42 (50.0)	-
Divorced	313 (15.7)	295 (15.4)	17 (20.2)	-
Widow	69 (3.5)	64 (3.3)	5 (6.0)	-
Educational level				0.872
High school	590 (29.5)	563 (29.4)	25 (29.8)	-
Bachelor	994 (49.7)	984 (49.6)	45 (53.6)	-
Master	313 (15.7)	303 (15.8)	10 (11.9)	-
PhD	85 (4.3)	82 (4.3)	3 (3.6)	-
Others	18 (0.9)	17 (0.9)	1 (1.2)	-
Total	2000	1916	84	-

**Table 2:** Distribution of the study's participants according to the Corona virus's infection history and C0VID-19 vaccine history and status. * P-value < 0.05; CI: confidence interval, NA: not applicable.

Items	Total n (%)	Vaccinated n (%)	Non-vaccinated n (%)	Vaccinated vs. non vaccinated OR (95% CI)
Do you suffer from chronic diseases like diabetes, coronary heart diseases, asthma, obesity, and gum disease?	-	-	-	-
Yes	893 (44.7)	844 (44.1)	47 (56.0)	0.62 (0.40-0.97)*
No	1107 (55.4)	1069 (55.9)	37 (44.0)	-
Do you have a history of COVID-19?	-	-	-	-
Yes	1174 (58.7)	1112 (58.2)	58 (69.0)	0.62 (0.39-0.99)*
No	826 (41.3)	799 (41.8)	26 (31.0)	-
If yes, what type of COVID-19 vaccine did you receive?	-	-	-	-
Pfizer-BioNTech	653 (32.7)	653 (32.7)	NA	-
Oxford-AstraZeneca	833 (41.7)	833 (41.7)	NA	-
Moderna	175 (8.8)	175 (8.8)	NA	-
Mixed	254 (12.7)	254 (12.7)	NA	-
Total	2000	1916	84	-

Moreover, regarding whether patients suffered from general post-vaccination symptoms, a response of "no" was 1.34 times higher than “yes” for the vaccinated group. Both results were not statistically significant (Table 3).

**Table 3:** Association between general post-vaccination symptoms and different COVID-19 vaccines Red indicates percentage within the group will add up to 100%. NA: not applicable. Ref: Reference where the odds ratio (OR)=1.

Vaccine	Vaccinated n (%)	Non vaccinated n (%)	Vaccinated versus non vaccinated
No	774 (40.6)	25 (30.9)	1.34 (0.63-2.82)
Pfizer-BioNTech	236 (30.5)	NA	-
Oxford- AstraZeneca	375 (48.4)	NA	-
Moderna	71 (9.2)	NA	-
Mixed	92 (11.9)	NA	-
Slightly	901 (47.3)	46 (56.8)	0.85 (0.42-1.70)
Pfizer-BioNTech	327 (36.3)	NA	-
Oxford- AstraZeneca	376 (41.7)	NA	-
Moderna	79 (8.8)	NA	-
Mixed	119 (13.2)	NA	-
Yes	231 (12.1)	10 (12.3)	Ref
Pfizer-BioNTech	88 (38.1)	NA	-
Oxford- AstraZeneca	78 (33.8)	NA	-
Moderna	24 (10.4)	NA	-
Mixed	41 (17.7)	NA	-
Total	1916	84	-

Periodontal tissue characteristics

A total of 223 vaccinated (11.7%) and six nonvaccinated participants (7.7%) experienced severe gingival bleeding. The odds ratio for gingival bleeding indicated no significant difference between the two groups (Table 4). Malodor, mobility, and tooth loss showed no statistical significance (Table 4).

**Table 4:** Post-vaccination side effects on periodontal tissues (gingival bleeding and malodor), in participants with chronic diseases and healthy participants. N: Never; SL: slightly; SV: severely. NA: not applicable. Ref: Reference, where the odds ratio (OR)=1.

Vaccine	Total N=2000, n (%)			Vaccinated n=1916, n (%)			Non vaccinated n=84, n (%)			Vaccinated vs. non vaccinated, n (%)		
	N	SL	SV	N	SL	SV	N	SL	SV	N	SL	SV
Gingival Bleeding	937 (47.1)	23 (41.4)	229 (11.5)	901 (47.1)	787 (41.2)	223 (11.7)	36 (46.2)	36 (46.2)	6 (7.7)	Ref	0.79 (0.35-1.80)	1.21 (0.76-1.93)
Malodor	943 (47.5)	799 (40.2)	244 (12.3)	913 (47.9)	765 (40.1)	230 (12.1)	30 (38.5)	34 (43.6)	14 (17.9)	Ref	0.78 (0.41-1.47)	0.56 (0.29-1.07)
Mobility	992 (50.0)	766 (38.6)	227 (11.4)	963 (50.5)	728 (38.2)	216 (11.3)	29 (37.2)	38 (48.7)	11 (14.1)	Ref	1.10 (0.56-2.19)	0.59 (0.29-1.20)
Tooth loss	1316 (66.3)	668 (33.7)	0	1266 (66.4)	640 (33.6)	0	50 (64.1)	28 (35.9)	0	Ref	0.96 (0.60-1.53)	NA

Moreover, there was a significant difference regarding the association between the severity of periodontal tissue symptoms and the type of vaccine (p-value < 0.05) (Table 5). The Oxford-AstraZeneca vaccine showed the most symptoms among all periodontal tissue symptoms (gingival bleeding, malodor, mobility, and tooth loss) (Table 5).

**Table 5:** Post-vaccination side effects on periodontal tissues in vaccinated patients are stratified by vaccine types. N: Never; SL: slightly; SV: severely; * P-value < 0.05.

Vaccine	Vaccinated n=1916, n (%)			P-value
	N	SL	SV	
Gingival Bleeding	-	-	-	<0.001*
Pfizer-BioNTech	404 (44.8)	196 (24.9)	43 (23.8)	-
Oxford-AstraZeneca	333 (37.0)	398 (50.6)	100 (44.8)	-
Moderna	72 (8.0)	79 (10.0)	24 (10.8)	-
Mixed	92 (10.2)	114 (14.5)	46 (20.6)	-
Malodor	-	-	-	-
Pfizer-BioNTech	404 (44.8)	197 (25.8)	49 (21.3)	-
Oxford-AstraZeneca	325 (35.6)	385 (50.3)	120 (52.2)	-
Moderna	63 (6.9)	85 (11.1)	27 (11.7)	-
Mixed	120 (13.1)	98 (12.8)	34 (14.8)	-
Mobility	-	-	-	<0.001*
Pfizer-BioNTech	409 (42.5)	175 (24.0)	49 (21.3)	-
Oxford-AstraZeneca	353 (36.7)	369 (50.7)	107 (49.5)	-
Moderna	68 (7.1)	83 (11.4)	24 (11.1)	-
Mixed	133 (13.8)	101 (13.9)	18 (8.3)	-
Tooth loss	-	-	-	0.011*
Pfizer-BioNTech	459 (36.3)	191 (29.8)	0	-
Oxford-AstraZeneca	544 (43.0)	286 (44.7)	0	-
Moderna	102 (8.1)	73 (11.4)	0	-
Mixed	161 (12.7)	90 (14.1)	0	-

## Discussion

Numerous studies have been carried out to evaluate the impact of COVID-19 on oral health. However, the potential link between COVID-19 vaccines and periodontal diseases has not been thoroughly discussed in the existing literature. Our results showed no statistically significant effect on periodontal conditions, although this effect varied between vaccines. The Oxford-AstraZeneca vaccine had potentially the highest effect compared with other vaccines.

Gingival bleeding is considered to be the first sign of periodontal inflammation and may indicate a potential interplay between COVID-19 vaccines and periodontal disease [22]. A recent study examined the potential connection between COVID-19 and periodontal disease, specifically through the role of IL-6 [22]. The study suggested that COVID-19 may worsen periodontal disease by increasing IL-6 production, leading to inflammation and tissue damage to the gums. Additionally, periodontal disease may worsen COVID-19 symptoms by contributing to higher IL-6 production. The authors recommend monitoring and treating periodontal disease in patients with COVID-19 to potentially decrease IL-6 levels and reduce the risk of severe COVID-19 outcomes [22].

In a case-control study, Anand et al. (2022) found a significant association between periodontitis and COVID-19. The study included 100 participants, 50 with periodontitis and 50 without, who were diagnosed with COVID-19. Patients with periodontitis had a higher risk of developing severe COVID-19 symptoms and higher levels of inflammatory markers, such as CRP. The study suggested that periodontitis may increase an individual’s susceptibility to COVID-19 and worsen its outcomes [23]. Good oral hygiene is important to reduce the risk of contracting and developing severe symptoms of COVID-19, which is also strongly suggested after the COVID-19 vaccination.

Klugar et al. (2021) revealed that the side effects of COVID-19 vaccines are generally mild and short-lived. They also reported that side effects include fatigue, headache, pain at the injection site, and fever. However, the impact of these vaccines on periodontal tissues has not been studied [24].

While the exact mechanism by which COVID-19 vaccines may impact periodontal tissues is not yet understood, certain hypotheses may explain the observed effects. One possibility is that the vaccines may cause an inflammatory response in the body, leading to increased bleeding in the gums and other effects on the periodontal tissue. This inflammation may also be linked to the different compositions of the vaccines and how they interact with the body [25,26].

Uehara et al. (2021) reported that the composition and diversity of the oral microbiome can undergo considerable changes following the COVID-19 vaccination. This alteration could have significant consequences for oral health. The vaccine’s immunomodulatory effects could plausibly be responsible for the changes in the oral microbiome. The findings demonstrate the importance of assessing the effect of COVID-19 vaccines on the oral microbiome, which could provide a likely explanation for the bad breath that some individuals may experience following vaccination. The vaccines manufactured by Moderna and Oxford-AstraZeneca had the most prominent correlation with bad breath (1.64% ± 0.48 and 1.61% ± 0.48, respectively). Comparatively, the Pfizer-BioNTech vaccine had the weakest relationship (1.38 ± 0.48) with malodor [27].

Our results highlight that the Pfizer-BioNTech vaccine had a greater impact on post-vaccination general side effects compared to the Moderna and Oxford-AstraZeneca vaccines. The study reports that the Pfizer-BioNTech vaccine had a mean value of 1.64 ± 0.48, while Moderna had a mean value of 1.59 ± 0.49, and Oxford-AstraZeneca had a mean value of 1.55 ± 0.5 (Table 3). Although it is not entirely clear why the Pfizer-BioNTech vaccine is associated with more side effects, the higher mRNA dose in the vaccine may be a factor. This finding may help healthcare providers inform patients about the potential side effects of the vaccine.

Similarly, a recent study conducted on 555 healthcare workers who received the Pfizer-BioNTech vaccine reported that the most common side effects were pain at the injection site, fatigue, headache, and myalgia [25]. These symptoms were mild to moderate and resolved within a few days. However, some participants reported severe symptoms such as fever and lymphadenopathy. Younger participants experienced more side effects than older participants, indicating that age may play a role in the severity of side effects.

It is crucial to understand that side effects are a normal immune response to the vaccine and are generally not cause for concern. However, they should be monitored and managed appropriately.

It is important to investigate the side effects experienced by participants with chronic diseases after vaccination, as it sheds light on the potential risks and benefits of vaccination for this population. The chronic diseases investigated in our study, including diabetes, coronary heart disease, asthma, obesity, and periodontal disease, can increase the risk of severe illness or death from COVID-19. Therefore, it is crucial to understand how individuals with these diseases might respond to vaccination. 

As different vaccines have become available, many people with chronic diseases, such as diabetes, asthma, coronary heart disease, and obesity, have had questions about how the vaccine could affect them. Our study was conducted to determine the general side effects of the COVID-19 vaccine in participants with chronic diseases compared to healthy participants. It was found that participants with chronic diseases experienced fewer general side effects, such as fatigue, headache, and fever, after the COVID-19 vaccination than healthy participants. This finding is significant because it suggests that people with chronic diseases can safely receive the vaccine without concern for severe side effects. Moreover, maintaining good oral hygiene is essential for overall health. Regular brushing and flossing can help prevent gingival bleeding and malodor. Additionally, individuals should consult with their healthcare providers if they have any oral health issues after the COVID-19 vaccination.

It is imperative to acknowledge and address certain limitations that may influence the interpretation of our results. First, recall bias poses a significant challenge, as participants may inaccurately recall periodontal inflammation signs and symptoms. Another limitation lies in the absence of a clinical examination to ascertain true signs and symptoms, as some of these conditions, such as oral malodor, could be attributed to various factors such as dental caries or other oral or medical health conditions. Moreover, the absence of a baseline assessment before vaccination complicates the determination of whether observed changes are linked to immunization. Standardized measurements are lacking due to self-reported conditions, which could introduce variability that may impact the reliability of the study's outcomes. Additionally, the study design is cross-sectional, which inherently limits the ability to establish causation between vaccination and periodontal conditions.

## Conclusions

The COVID-19 vaccines might not have a specific direct impact on periodontal tissues. However, our study found that more than half of the individuals who received COVID-19 vaccinations encountered nonspecific oral manifestations, such as bleeding gums and oral malodor. However, people should not avoid vaccination due to concerns with oral health because the benefits of vaccination outweigh any potential side effects. Individuals should be aware of any changes or symptoms they may experience after vaccination and should report them to their healthcare provider if necessary. Further research is needed on the effects of COVID-19 vaccines on oral health, particularly periodontal diseases.

## Figures and Tables

**Figure 1:**
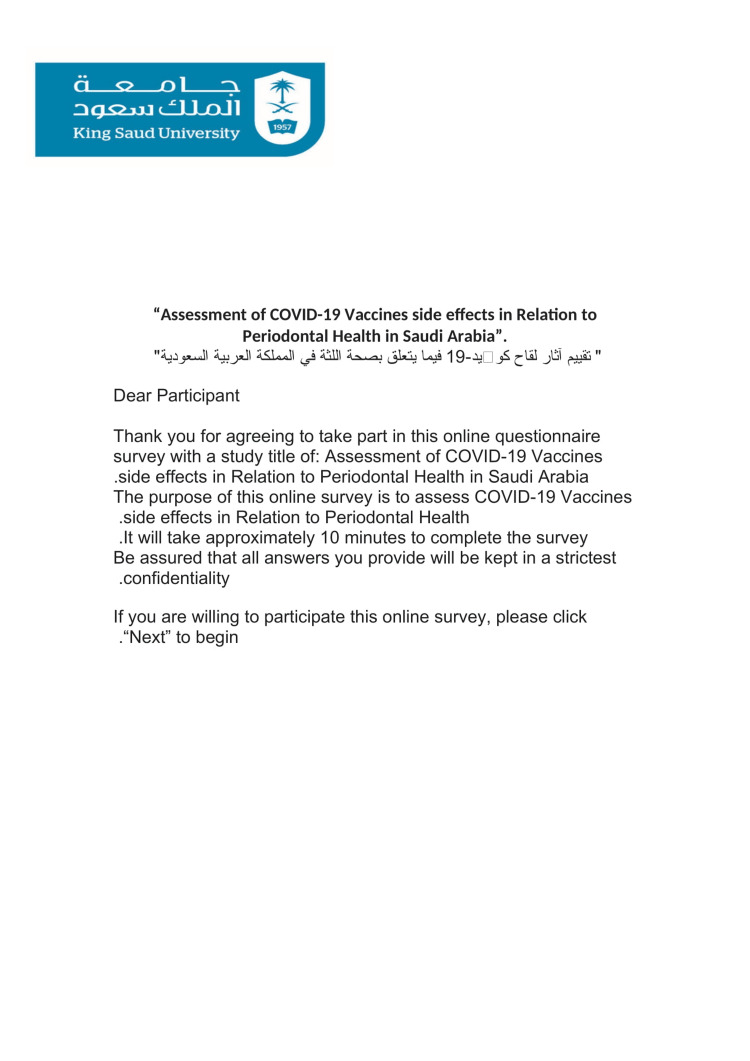
Questionnaire first page

**Figure 2:**
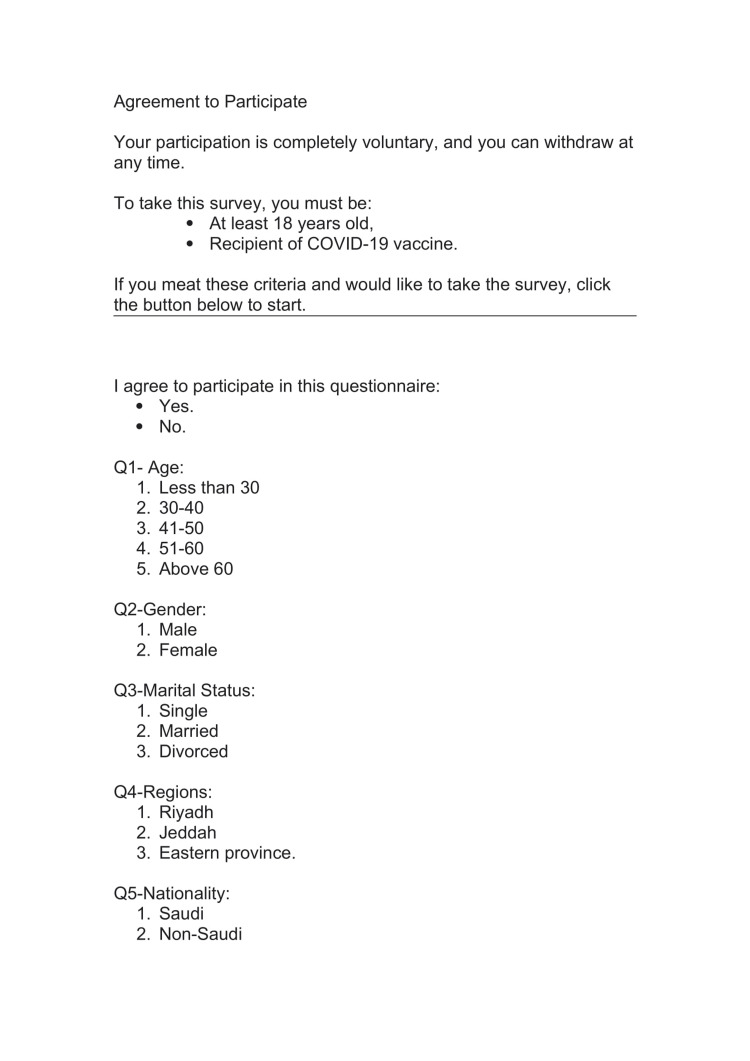
Questionnaire second page.

**Figure 3:**
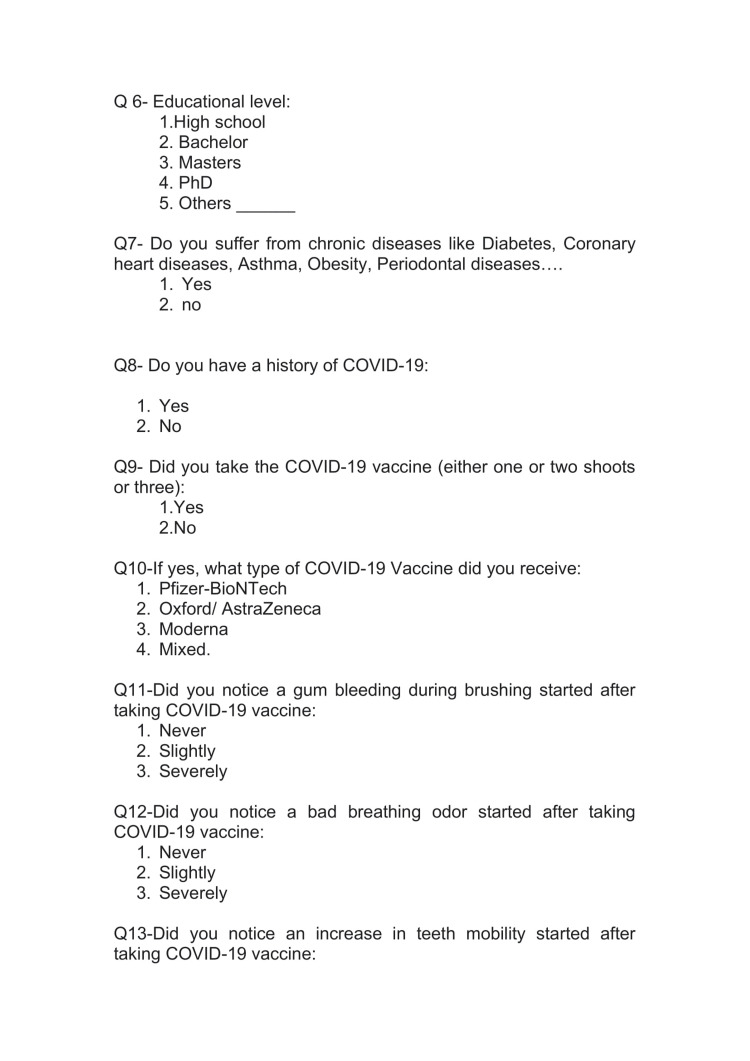
Questionnaire third page.

**Figure 4:**
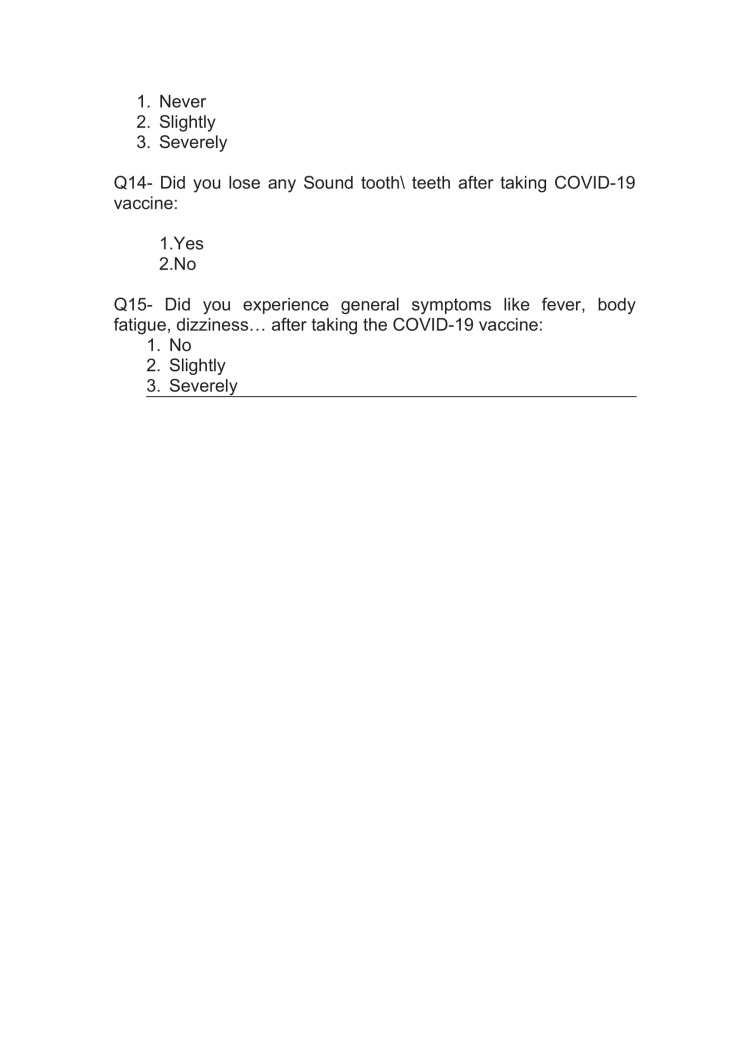
Questionnaire fourth page.
